# Pathogen-Reduced Low-Titer Group O Whole Blood for Managing Massive Blood Loss in Prehospital and Early Hospital Settings: An In Vitro Study

**DOI:** 10.3390/jcm14176292

**Published:** 2025-09-05

**Authors:** Ekaterina Sherstyukova, Julia Semenova, Snezhanna Kandrashina, Alina Bogdanova, Ilya Vinogradov, Vladimir Inozemtsev, Mikhail Shvedov, Alexander Grechko, Maxim Dokukin, Artem Kuzovlev, Elena Klychnikova, Andrey Bulanov, Alexander Kostin, Viktoria Sergunova

**Affiliations:** 1 Federal Research and Clinical Center of Intensive Care Medicine and Rehabilitology, V.A. Negovsky Research Institute of General Reanimatology, Moscow 107031, Russiavika_23s82@mail.ru (V.S.); 2 Moscow Department of Health, Sklifosovsky Research Institute for Emergency Medicine, Moscow 129090, Russiakostinai@sklif.mos.ru (A.K.)

**Keywords:** AFM, leukoreduced whole blood, LTOWB, massive blood loss, membrane elasticity, pathogen inactivation, pathogen reduction, red blood cells

## Abstract

**Background/Objectives**: Leukoreduced low-titer group O whole blood (LTOWB) is a promising option for early transfusion in massive hemorrhages, by providing red blood cells (RBCs), plasma, and platelets in a single unit. This study aimed to assess the effects of pathogen reduction (PR) on the LTOWB quality and its suitability for emergency transfusions, including its post-thaw RBC usability. **Methods**: Whole blood from 24 donors was processed and analyzed over seven days, including post-thaw assessments. Hematological and coagulation parameters, along with atomic force microscopy, were used to assess the RBCs’ morphology, cytoskeletal integrity, and nanomechanical properties. **Results**: The PR reduced the leukocyte content while preserving platelet counts at the level of the control group. Although certain clotting factors, such as fibrinogen (*p* < 0.001) and factor VIII (*p* < 0.001), were diminished after the PR, thromboelastometry results remained within reference ranges, indicating a maintained hemostatic potential. Morphological changes in RBCs were observed, but the membrane elasticity remained stable throughout storage and after thawing, indicating preserved macromechanical properties essential for hemostasis. **Conclusions**: These findings demonstrate that LTOWB treated with partial a leukoreduction and PR maintains functional and structural integrity, supporting its potential utility as a safe and effective transfusion product for managing acute blood loss.

## 1. Introduction

Hemorrhagic shock is one of the most common causes of mortality in trauma cases. Globally, severe trauma accounts for approximately 4.4 million deaths annually—about 8% of all deaths—with 35–40% of these cases attributed to uncontrolled bleeding [1]. The majority of patients with hemorrhagic shock die before arriving at the hospital, highlighting the importance of rapid and effective prehospital and early hospital resuscitation [2,3]. In this context, low-titer group O whole blood (LTOWB) is gaining renewed interest as a universal and balanced transfusion solution for the primary correction of massive blood loss—whether traumatic or non-traumatic—in patients regardless of their blood type and Rh factor [4].

LTOWB is considered safe for both adults and children [5,6] and offers advantages over component therapy by providing red blood cells (RBCs), clotting factors, plasma, and cold platelets (PLTs) in a single unit. It contains fewer anticoagulants and additives, which may reduce hemodilution during massive transfusions [7,8,9], and its use reduces the strain on donor resources [10,11].

Historically, whole blood (WB) was transfused within 48 h without leukoreduction [12]. While leukoreduction is standard in many countries, its necessity for all blood products remains debated [10,12,13]. In the Russian Federation, there are two options for the procurement of preserved blood: with and without the use of leukoreduction [14,15]. At the same time, harvesting methods using platelet-preserving leukocyte filters, which reduce the number of leukocytes but preserve PLTs, have not yet been approved for use. Thus, this is the first study in Russia applying this novel processing method designed as an alternative to platelet-sparing leukoreduction filters.

Our study presents a modified LTOWB preparation protocol that combines the partial leukoreduction of the LTOWB and pathogen reduction (PR), aiming to improve the safety and effectiveness of whole blood transfusions in prehospital and early hospital settings. The partial leukoreduction removes a substantial portion of leukocytes while retaining functional platelets, which—together with fibrinogen and factor XIII—play a central role in emergency hemostasis during the correction of traumatic coagulopathy.

Pathogen reduction helps minimize cytomegalovirus transmission [16,17,18,19] and the alloimmunization risk [10,20,21,22], critical in repeated transfusions. However, PR may reduce platelet function and lower the hemostatic potential, raising concerns about its impact on transfusion efficacy during massive bleeding. The safety of WB transfusions containing large numbers of donor leukocytes continues to be debated, as the risk of immune complications in recipients persists [10,23].

In this study, we aimed to evaluate the impact of PR on the structural and functional properties of LTOWB and to determine its suitability for emergency transfusions in hemorrhagic shock. Additionally, we examined whether thawed RBCs remain viable for further use as a blood component. To achieve this, we applied a comprehensive approach including, on the one hand, using atomic force microscopy (AFM) to examine the morphological and nanostructural integrity and the cytoskeletal structure of RBCs and to assess the membrane elasticity, and, on the other hand, the analysis of the coagulation capacity and clotting factor preservation. This allowed for a thorough assessment of the quality of donor blood products.

## 2. Materials and Methods

### 2.1. Blood Product Preparation

WB donations from 24 healthy male volunteers (blood group O, low anti-A/B antibody titers < 1:64) were used in this study. All donors provided written informed consent, and each donation was tested for transfusion-transmissible infectious diseases. This study was approved by the Ethical Committees of the N.V. Sklifosovsky Research Institute for Emergency Medicine (Protocol No. N4/2024, 27 August 2024) and the Federal Research and Clinical Center of Intensive Care Medicine and Rehabilitology (Protocol No. 2/20, 10 June 2020). All procedures were conducted in accordance with the Declaration of Helsinki.

Donor blood with a 450 mL ± 10% volume was collected using the Reveos Select Blood Bag Set (Terumo BCT, Lakewood, CO, USA) plastic vacuum containers according to the manufacturer’s instructions. Additionally, 63 mL of citrate solution (CPD-1, citrate–phosphate–dextrose) was used as anticoagulant. The total volume of whole blood was ~513 mL.

LTOWB units were stored at +4–6 °C for up to 7 days and subsequently cryopreserved. In accordance with the current regulations of the Russian Federation, red blood cells must be frozen for further use no later than 168 h post-donation, i.e., by day 7 of storage. Therefore, in our study, the storage period prior to cryopreservation was limited to 7 days. To optimize resource utilization and avoid the disposal of valuable material, all LTOWB units were fractionated on day 7 and transferred to the cryobank for further storage (8–14 days). To assess the suitability of pathogen-reduced red blood cells after cryopreservation, samples were thawed and evaluated for structural and functional integrity.

The Valeri method was used to freeze RBCs. The whole blood was transferred to an ethyl vinyl acetate bag. Before freezing, the blood unit was centrifuged to separate the RBCs, followed by standard glycerolization using the ACP 215 device (Haemonetics, Braintree, MA, USA). Glycerin was added to the RBCs to a final concentration of 40%. The RBC bags then underwent cryopreservation in MACO BIOTEC containers (Macopharma, Tourcoing, France) and were stored in liquid nitrogen. A Barkey device (Barkey GmbH & Co. KG, Leopoldshöhe, Germany) was used for thawing. The RBCs were thawed using standard technology with deglycerolization, which was also performed on the ACP 215 device, and SAGM was used as the resuspension solution.

This study included two groups: a control group (n = 12) and a pathogen reduction group (n = 12).

The control group consisted of partially leukoreduced stored blood, processed using the Reveos automated WB processing system (off-label), with platelet preservation.

The PR group included partially leukoreduced WB processed with the Reveos system, followed by pathogen reduction using the Mirasol system (Terumo BCT, Lakewood, CO, USA). This method involves photochemical inactivation of pathogens and leukocytes using UV light and riboflavin. Briefly, 35 mL of riboflavin (500 µmol/L) was added to the WB in an irradiation bag, mixed, and exposed to 80 J/mL of UV light. Samples were collected after inactivation for further analysis.

Samples (3–5 mL) were aseptically collected on days 1 (D1) and 7 (D7) and after thawing using TSCD II sterile connectors (Terumo BCT, Lakewood, CO, USA).

### 2.2. Laboratory Investigations

The Sysmex XN-350 hematology analyzer (Sysmex Corporation, Kobe, Japan) was used to perform complete blood counts, measuring hemoglobin (HGB), hematocrit (HCT), RBCs, white blood cells (WBCs), and PLTs.

The ROTEM delta system (Tem Innovations GmbH, Munich, Germany) was used to assess blood coagulation using the NATEM test, in which 20 µL of 0.2 M CaCl_2_ was added to each sample as a recalcification agent, without the use of an external activator [24,25]. Measured parameters included the following: clotting time (CT), clot formation time (CFT), alpha angle, amplitude recorded at 20 min (A20), maximum clot firmness (MCF), and lysis index at 30 min (LI30).

After centrifuging (3000 rpm, 12 min) the samples to separate the plasma, analysis was performed on an ACL TOP 750 automatic coagulometer (Werfen, Bedford, MA, USA). The analyzer measured prothrombin time (PT), international normalized ratio (INR), activated partial thromboplastin time (aPTT), thrombin time (TT), fibrinogen, factor VIII, factor XIII, von Willebrand factor—antigen and ristocetin cofactor activity (RCO)—as well as the level of hemolysis in the samples.

Free hemoglobin, required for calculating the percentage of hemolysis, was measured using the HemoCue Plasma/Low Hb analyzer (HemoCue AB, Ängelholm, Sweden).

Atomic force microscopes NTEGRA Prima and NTEGRA BIO (NT-MDT SI, Moscow, Russia) were used to assess RBC morphology, membrane nanostructure, cytoskeletal integrity, and elasticity. Imaging was performed in semi-contact mode using NSG01 cantilevers (tip radius 10 nm). Image processing was performed with FemtoScan Online software 2.3.239 (5.2) (Advanced Technologies Center, Moscow, Russia) and Image Analysis 3.5 (NT-MDT SI, Moscow, Russia), and nanostructure analysis was performed with Gwyddion 2.65 (Czech Metrology Institute, Brno, Czech Republic) [26].

For nanostructural profiling, cross-sectional profiles were generated from a 2.5 × 2.5 μm^2^ scan area, corresponding to one-fourth of the total image field. The maximum wave height (W_max_) was measured from these profiles, denoted as W_y_ = W_max_. This parameter reflects surface waviness—a low-frequency component that characterizes the overall topography of the membrane surface.

To evaluate Young’s modulus (E), force spectroscopy was conducted using cantilevers from the SD-R150-T3L450B-10 series (Nanosensors, Neuchâtel, Switzerland), featuring a tip radius of 150 nm and a force constant of 1 N/m.

### 2.3. Statistical Analysis

Statistical analysis was performed using OriginPro2019 (OriginLab Corporation, Northampton, MA, USA). Data distribution was assessed with the Shapiro–Wilk test. Results are presented as mean ± standard deviation (SD) for normally distributed variables and as median (Me) with interquartile range (Q1; Q3) for non-normally distributed variables. Comparisons between independent samples were performed using the Mann–Whitney U test, a nonparametric method appropriate for analyzing differences in data that do not follow a normal distribution. For paired samples, the Wilcoxon signed-rank test was applied. Statistical significance was set at *p* < 0.05 (two-sided).

## 3. Results

Our primary objective was to assess the suitability of RBCs within the modified blood product, as well as its capacity to effectively correct the coagulopathy in the context of its anticipated use in patients with a massive hemorrhage. For this purpose, in the first stage of this study, we analyzed hematological parameters, viscoelastic test results, and levels of clotting factors.

In accordance with the current regulations of the Russian Federation, the storage period for the product was limited to seven days. These regulations stipulate that whole blood must be fractionated and its components frozen no later than 168 h (7 days) after donation.

### 3.1. Changes in Hematologic Parameters

**Figure 1:**
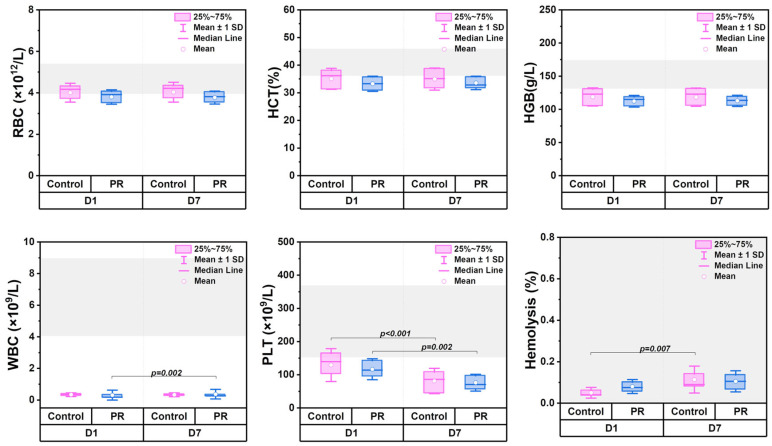
Box plots comparing hematological parameters in control (n = 12) and PR (n = 12) groups on day 1 (D1) and day 7 (D7) of storage. Gray area shows reference ranges. Parameters include hematocrit (HCT), hemoglobin (HGB), hemolysis, platelets (PLTs), red blood cells (RBCs), and white blood cells (WBCs). Statistical significance is indicated as the exact *p*-value.

Hematological parameters on days 1 and 7 of storage are shown in Figure 1. The RBC count and hematocrit remained stable in both groups. Hemoglobin levels were 118 ± 13 g/L (60 g/dose) in the control and 112 ± 8 g/L (58 g/dose) in the PR group. The partial leukoreduction with the Reveos system (off-label) reduced WBCs to 0.36 (0.25; 0.37) × 10^9^/L (D1) and 0.33 (0.25; 0.37) × 10^9^/L (D7) in the control and to 0.18 (0.14; 0.33) × 10^9^/L (D1) and 0.26 (0.21; 0.36) × 10^9^/L (D7) in the PR group. PLTs decreased from (129.2 ± 49.4) × 10^9^/L (control D1) to (81.0 ± 38.3) × 10^9^/L (control D7) and from (116.5 ± 31.7) × 10^9^/L (PR D1) to (76.1 ± 25.5) × 10^9^/L (PR D7), corresponding to 0.66 × 10^11^/dose and 0.39 × 10^11^/dose. No significant differences were observed between groups (*p* > 0.5). Hemolysis did not exceed the acceptable threshold of 0.8% [27] on either D1 or D7.

### 3.2. Measures of Coagulation Parameters

**Figure 2:**
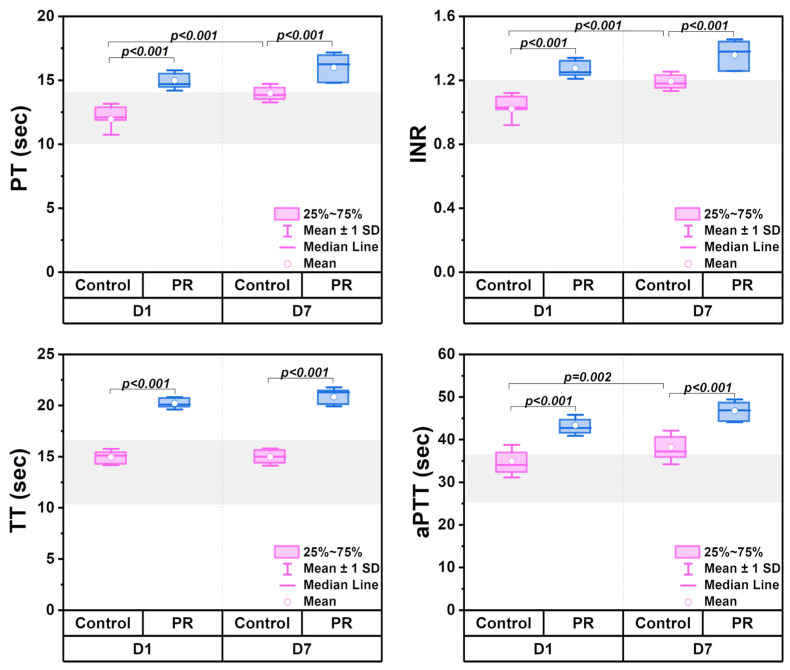
Box plots showing coagulation parameters in control (n = 12) and PR (n = 12) groups on D1 and D7 of storage. Parameters include activated partial thromboplastin time (aPTT), international normalized ratio (INR), prothrombin time (PT), and thrombin time (TT). The gray area indicates reference ranges. Statistical significance is indicated as the exact *p*-value.

Coagulation testing revealed an increase in the PT, INR, and aPTT during storage (Figure 2). In the control group, mean values remained close to reference ranges: the PT (10–14 s), INR (0.8–1.2), TT (10.3–16.6 s), and aPTT (25.1–36.5 s). PR led to a significant prolongation of the PT, INR, and aPTT compared to controls. The statistical significance is indicated in the box plots.

**Figure 3:**
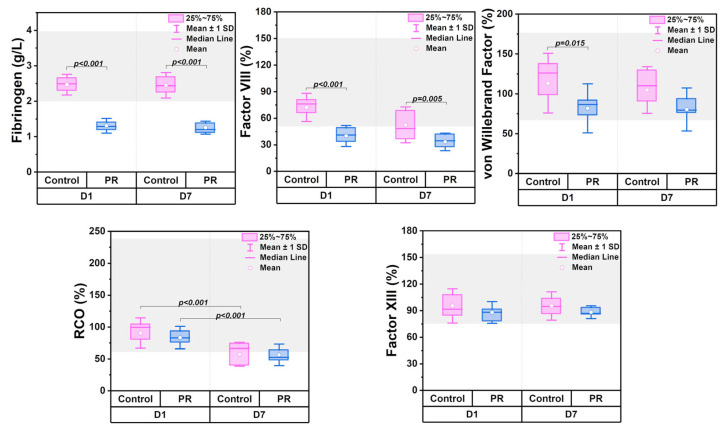
Box plots comparing clotting factor levels in control (n = 12) and PR (n = 12) groups on D1 and D7 of storage. Parameters include factor VIII, factor XIII, fibrinogen, ristocetin cofactor activity (RCO), and von Willebrand factor antigen. The gray area represents reference ranges. Statistical significance is indicated as the exact *p*-value.

Figure 3 shows the changes in clotting factors, with the gray area indicating reference ranges. The PR significantly reduced fibrinogen levels (*p* < 0.001), with mean values of 1.3 ± 0.2 g/L on D1 and 1.2 ± 0.2 g/L on D7. Factor VIII levels declined due to both the PR and storage time. The PR also led to a significant reduction in von Willebrand factor levels on D1 (*p* = 0.015), while the storage time had no effect. The RCO decreased over time (*p* < 0.001) but was not affected by the PR. Factor XIII levels remained stable and were unaffected by either the PR or storage duration (*p* > 0.05).

**Figure 4:**
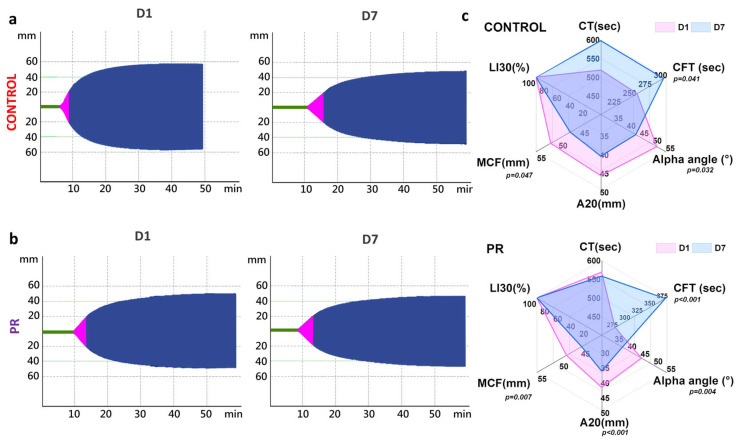
Changes in ROTEM parameters in control (n = 12) and PR (n = 12) groups during storage. (**a**,**b**) Representative thromboelastograms for control and PR groups on D1 and D7 of storage. (**c**) Radar plots comparing relative changes in ROTEM parameters, including alpha angle, amplitude at 20 min (A20), clot formation time (CFT), clotting time (CT), lysis index at 30 min (LI30), and maximum clot firmness (MCF). Exact *p*-values between D1 and D7 are indicated.

At the same time, thromboelastometry parameters remained within reference ranges and showed no significant differences between groups on D1 (*p* < 0.05). Representative thromboelastograms for control and PR groups on D1 and D7 are shown in Figure 4a,b. The CT increased to 600 ± 55 s in the control group during 7 days of storage (Figure 4c). The MCF decreased by 4 mm in both groups by D7. The LI30 remained unchanged. The alpha angle and A20 decreased by 8° and 5 mm by D7, respectively. On D7, differences between groups were noted for the alpha angle (*p* = 0.46) and A20 (*p* = 0.004). The CFT exceeded the reference range (60–150 s) in both groups. In the control group, the CFT increased from 215 (150; 277) s to 283 (250; 326) s (*p* = 0.041), and in the PR group it increased from 273 (247; 289) s to 372 (295; 469) s (*p* < 0.001), with a between-group difference on D7 (*p* = 0.032).

### 3.3. Erythrocyte Morphology and Young’s Modulus

In the second stage, the erythrocyte morphology, elastic properties, and cytoskeleton structure were studied using AFM.

**Figure 5:**
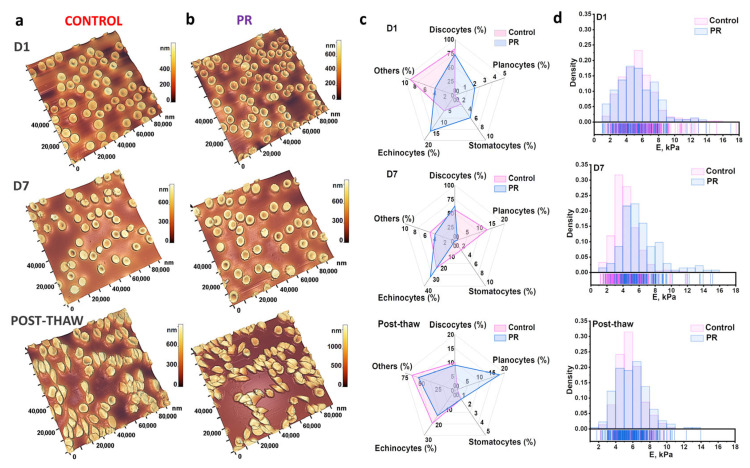
Morphological and mechanical changes in RBCs during storage and after thawing. (**a**) AFM 3D images of RBCs from the control group on D1, on D7, and post-thaw. (**b**) AFM 3D images of RBCs from the pathogen reduction (PR) group on D1, on D7, and post-thaw. (**c**) Radar plots showing the distribution (%) of RBC shapes (discocytes, echinocytes, planocytes, stomatocytes, others) in control and PR groups on D1, on D7, and after thawing. (**d**) Histograms with rug plots illustrating the distribution of Young’s modulus (E) in control and PR groups across all time points.

The RBC morphology changed over time in both groups. AFM was used to examine PR-related alterations in the cell shape in more detail. Figure 5b shows representative 100 × 100 μm^2^ AFM images of RBC smears on D1 and D7 and after thawing.

Five RBC forms were identified: discocytes, planocytes, stomatocytes, echinocytes, and others. The morphology was assessed daily and visualized as radar plots (Figure 5c). On D1, discocytes predominated: 83 ± 7% in control and 73 ± 8% in PR. The PR increased echinocytes to 16 ± 5%. By D7, control samples showed 56 ± 7% discocytes, 24 ± 5% echinocytes, 13 ± 5% planocytes, and 2 ± 1% stomatocytes. PR samples on D7 contained 63 ± 5% discocytes and 32 ± 6% echinocytes. Discocytes and planocytes are generally considered functionally competent shapes associated with optimal deformability and oxygen transport. In contrast, echinocytes and stomatocytes may indicate membrane instability, oxidative stress, or early signs of damage that can compromise the microcirculatory flow.

After D7 of storage, both the control and PR samples were fractionated, and the erythrocytes were subsequently frozen. As frozen blood is a critical resource for blood banks, particularly in emergencies, but is used only as a blood component [28,29], it was essential to assess the RBC quality after thawing (Figure 5). The post-thaw analysis showed a dominance of “other” cell types: 65 ± 7% in the control and 55 ± 8% in the PR. Discocytes decreased to 10 ± 3% (control) and 9 ± 3% (PR). Echinocytes were 22 ± 7% and 17 ± 5%, respectively. Stomatocytes were 1 ± 0.5% in both groups. Planocytes increased in the PR to 18 ± 4% (vs. 1 ± 0.5% in control). Although the post-thaw samples showed a significant decrease in discocytes, the presence of planocytes alongside moderate Young’s modulus values indicates that some cells may still be sufficiently deformable for clinical use. However, this requires further validation.

Young’s modulus was measured to evaluate the cell deformability [14,30]. Figure 5d shows the distribution of Young’s modulus for each sample on all experimental days, presented as histograms with rug plots. Throughout storage and after thawing, E remained stable at 5 ± 2 kPa, with no significant difference between control and PR groups (*p* > 0.05).

### 3.4. The Evaluation of the Cytoskeleton and Nanostructure of Erythrocyte Membranes

**Figure 6:**
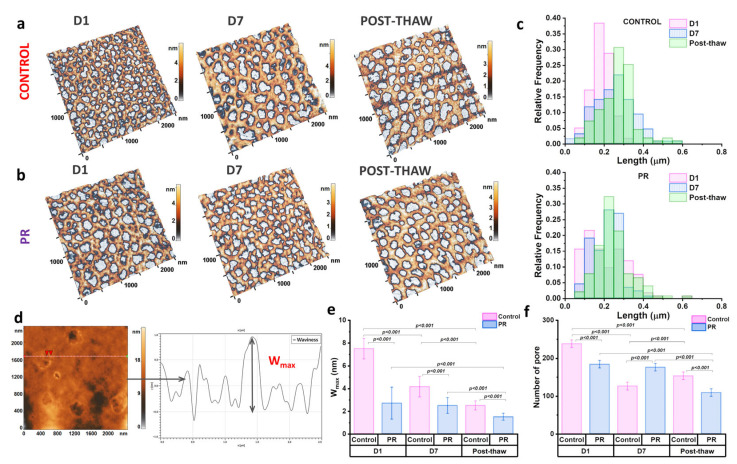
Changes in erythrocyte cytoskeletal structure and membrane nanostructure during storage and after thawing. (**a**,**b**) AFM 3D images of cytoskeletal fragments (2.5 × 2.5 μm^2^) in control (**a**) and PR (**b**) groups on D1, on D7, and after thawing. (**c**) Histograms showing the distribution of pore lengths in control and PR groups at all time points. (**d**) Schematic representation of nanostructure parameter measurements, including maximum wave height (W_max_). (**e**) Graph showing changes in W_max_ in both groups during storage and after thawing. (**f**) Graph showing changes in pore number in control and PR groups on D1, on D7, and post-thaw. Statistical significance is indicated as the exact *p*-value.

The cytoskeletal state plays a key role in regulating the nanostructural organization of the cell membrane, thereby affecting the overall cell morphology. To investigate these relationships, a detailed analysis of the erythrocyte cytoskeleton and membrane nanostructure was performed. Typical 2.5 × 2.5 μm^2^ membrane areas are presented in Figure 6a,b. In the AFM images, protein filaments are visualized as brown areas, while light-gray regions correspond to voids referred to as membrane pores.

The storage time and PR influenced the cytoskeletal structure. The pore size and number were quantitatively assessed (Figure 6c,f). In the control group, storage led to an increase in pore length from 0.18 ± 0.05 μm on D1 to 0.25 ± 0.09 μm on D7 (*p* < 0.0001). After thawing, the pore length remained unchanged at 0.25 ± 0.08 μm. In the PR group, the mean pore length was not affected by storage (0.21 ± 0.11 μm); however, it significantly increased after thawing to 0.27 ± 0.08 μm (*p* < 0.001).

In addition, changes in the pore number paralleled alterations in the pore length. During storage, the number of pores decreased in the control group, while remaining stable in the PR group (Figure 6f). Following thawing, both groups exhibited a reduction in the pore number, to 109 ± 12 in the control and 153 ± 10 in the PR group (*p* < 0.001).

Figure 6d schematically illustrates the measurement of nanostructural parameters, with profiles drawn across a 2.5 × 2.5 μm^2^ region, representing one-quarter of the total scanned area. The W_max_, defined as the peak-to-valley distance along a profile line, reflects the vertical amplitude of membrane surface features driven by the underlying cytoskeleton. As shown in Figure 6e, the W_max_ decreased over storage in the control group from 7.5 ± 0.9 nm to 4.2 ± 0.9 nm. In contrast, the W_max_ in the PR group remained relatively unchanged at 2.5 ± 0.7 nm. After thawing, the W_max_ decreased in both groups by 1.6 times. Differences between the groups were statistically significant (*p* < 0.001).

## 4. Discussion

Leukoreduced preserved blood represents a potentially effective transfusion medium for managing acute massive hemorrhages. The current standard in blood component processing is total leukoreduction, a procedure significantly reducing post-transfusion reaction risks [18,31,32].

However, PLTs are significantly reduced during leukoreduction [19], making it impossible to use such blood to correct massive blood loss [18,19]. On the other hand, the use of non-leukoreduced blood overloads the potential recipient with donor leukocytes and significantly increases the risk of post-transfusion reactions [33,34].

The search for an optimal blood preparation method remains a relevant challenge [35,36,37,38,39]. In this study, we applied a modified approach to prepare LTOWB. The control group underwent leukoreduction using the Reveos automated processing system, while the PR group received additional pathogen inactivation with riboflavin and UV light.

Riboflavin combined with UV light (280–360 nm) induces nucleic acid damage via electron transfer, singlet oxygen generation, and hydroxyl radical formation. This process can occur even in the absence of oxygen [40]. Riboflavin/UV-based pathogen reduction has shown efficacy against a wide range of pathogens, including bacteria and viruses, in preclinical studies of PLTs and plasma [41,42,43].

As a result of the pathogen inactivation, the number of platelets remained comparable to the control group on each day of storage. At the same time, a significant decrease in the leukocyte count was observed in both groups, with a more pronounced reduction in the PR group. The lower leukocyte content compared to the control further highlights the efficiency of the cell removal and indicates the enhanced potential safety of this product for transfusion.

Importantly, hemoglobin levels in each sample remained stable throughout the storage period and were close to reference values, indicating preserved erythrocyte integrity. Although slightly below standard donor levels, these values fall within clinically acceptable thresholds for transfusion and are consistent with a preserved cell content and viability, and hemoglobin concentrations of 118 ± 13 g/L (control) and 112 ± 8 g/L (PR) per unit (equivalent to 60 g/dose and 58 g/dose, respectively) are considered sufficient for effective transfusions [44]. Hemolysis levels in both groups were minimal and did not exceed the acceptable threshold of 0.8%, further confirming the product quality.

According to the literature, most blood coagulation proteins remain largely unaffected during storage at 4 °C [7,45]. In our study, the pathogen reduction reduced coagulation factors, particularly affecting fibrinogen and factor VIII (Figure 3).

However, during storage, these parameters remained at a consistent level. The von Willebrand factor is crucial in protecting factor VIII from proteolytic degradation [46]. The possible reduction in the von Willebrand factor due to pathogen reduction may have led to a decrease in factor VIII below reference values. On the other hand, the prolonged PT, INR, and aPTT also confirmed that the coagulation factors were reduced. Equally important is the consideration of ADAMTS13, a key regulator of the von Willebrand factor activity. ADAMTS13 is responsible for the cleavage of ultra-large von Willebrand factor multimers, thereby regulating thrombus formation and the microvascular flow. Since the pathogen reduction affected von Willebrand factor levels in our study, it remains unclear whether the ADAMTS13 activity is also influenced. The von Willebrand factor/ADAMTS13 axis has been shown to play a critical role in thrombotic disorders and in the prognosis of ischemic stroke outcomes [47]. Including ADAMTS13 in future analyses could provide a more comprehensive understanding of the hemostatic potential of PR-treated LTOWB.

Despite the reduction in certain coagulation factors and platelet levels in the PR group, thromboelastometry parameters, MCF and LI30, remained within reference ranges, indicating a preserved global hemostatic potential [12,48,49]. However, ROTEM does not assess the platelet aggregation or individual factor activity, and further functional studies are needed to confirm these findings. This is critically important, as platelets, along with fibrinogen and factor XIII, play a key role in clot formation during the correction of traumatic coagulopathy, providing urgent hemostasis and preventing further blood loss, particularly in emergency transfusion settings. In this context, the maintained ROTEM profiles suggest that, despite the observed reduction in certain clotting factors, the overall clotting capacity of the product remains adequate for clinical use. This result suggests the potential use of such blood in cases of massive blood loss; however, this hypothesis requires confirmation through in vivo studies.

In earlier work, Pidcoke et al. [50] explored the feasibility of pathogen-reduced whole blood for massive hemorrhage management, while Tomas et al. [10] investigated the effects of sequential leukoreduction and pathogen inactivation on clot formation. It was shown that both leukoreduction and pathogen reduction affect the functional properties of platelets. A significant decrease in the platelet count, clot density, and fibrinogen levels was observed in the group where stored blood underwent sequential leukoreduction followed by PR. However, these studies did not examine the ultrastructural and mechanical consequences for erythrocytes. The lack of a nanomechanical analysis in prior research limits our understanding of how these interventions affect erythrocyte integrity and functionality.

Our study addresses this gap by employing AFM to evaluate the erythrocyte morphology and mechanical properties throughout storage. Notably, pathogen reduction resulted in a higher percentage of echinocytes on D7 of storage. Echinocytosis may reflect early membrane alterations; however, the echinocyte fraction remained moderate and likely composed of reversible forms, as supported by the preserved elasticity and cytoskeletal architecture. Interestingly, despite the change in shape, the elasticity modulus of the erythrocyte membranes remained approximately at the same level (5 kPa). These values fall within the expected physiological range for healthy, deformable RBCs (typically 4–8 kPa) [51]. This global mechanical indicator may not fully capture local membrane remodeling, but its stability suggests that essential deformation properties of RBCs were maintained. The preservation of elastic properties is crucial for erythrocytes as they traverse capillaries and for effective oxygen delivery [52]. The membrane retained its overall elasticity and mechanical strength because there were no significant changes in cytoskeletal proteins, such as spectrin and actin, which support the membrane’s mechanical properties [53]. This explains why erythrocytes can change shape while keeping Young’s modulus unchanged.

**Figure 7:**
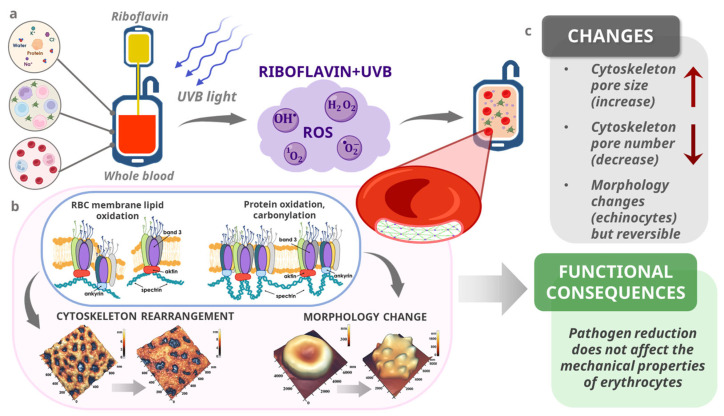
Suggested molecular pathway of oxidative and structural changes in erythrocytes following riboflavin and UV exposure. (**a**) Effects of Riboflavin/UVB on whole blood. (**b**) The effect of oxidative stress on cytoskeleton rearrangement and erythrocyte morphology. (**c**) Changes observed in the study and possible functional consequences.

Although this study did not directly evaluate molecular signaling pathways or protein expression levels, the observed alterations in the erythrocyte membrane structure and mechanics can be interpreted in the context of cytoskeletal remodeling at the molecular level. In Figure 7, we have shown the possible molecular mechanism of erythrocyte changes after pathogen reduction. Previous studies have shown that oxidative stress and UV-based treatments can induce protein crosslinking or degradation or the detachment of membrane-associated proteins (Figure 7a) [54,55,56]. Riboflavin/UV pathogen reduction, in particular, generates singlet oxygen and hydroxyl radicals that may target sulfhydryl groups and tyrosine residues within cytoskeletal proteins, leading to conformational changes without immediate functional disruption [57]. Oxidative stress and reactive oxygen species can cause the oxidation of membrane lipids and the oxidation of proteins and their carbonylation, which in turn can lead to changes in the cytoskeleton structure and cell shapes, as shown in Figure 7b. The stability of Young’s modulus, despite changes in the cell morphology and nanostructure, suggests that the essential components of the membrane-supporting cytoskeleton—particularly the spectrin, actin, and ankyrin complexes—may remain functionally undamaged during pathogen reduction and storage (Figure 7c). The preservation of the W_max_ and pore number in the PR group, compared to the control, may indicate a protective or stabilizing effect on these structures. Additionally, changes in the number and dimensions of membrane pores observed in the AFM analysis may reflect molecular-scale alterations in the anchoring of spectrin–actin junctions or the rearrangement of lipid–protein microdomains. These nanostructural changes could represent early stages of membrane aging or adaptation, which precede gross mechanical failure.

The consistent preservation of membrane elasticity across all time points further suggests that the mechanical resilience of the erythrocyte is not solely dependent on morphology but is maintained through a robust molecular architecture. These findings highlight the need for future studies combining AFM with molecular assays (e.g., immunolabeling, Western blotting, or mass spectrometry) to investigate specific post-translational modifications or oxidative damage to cytoskeletal and membrane proteins during storage and pathogen reduction.

Since frozen blood serves as an essential strategic reserve for blood banks in conditions of limited access to fresh components [58], our study also included an assessment of RBCs after thawing. For this purpose, all LTOWB samples were separated into fractions after day 7 of storage and frozen, then the units of RBCs were thawed. Although the post-thaw samples showed a marked reduction in discocytes and the presence of ghosts, the preserved Young’s modulus and the appearance of planocytes suggest that a subset of RBCs may retain sufficient deformability. Nevertheless, the clinical relevance of these cells must be evaluated in functional and in vivo studies. In the PR group, an increase in the pore length and a decrease in the pore number were observed. Additionally, the maximum waviness parameter was lower than before freezing, which may reflect a smoothing of the membrane topography; however, it could also indicate the loss of fine structural features, requiring further investigation to determine whether this is a stabilizing or damaging effect.

These findings support the potential use of PR-treated LTOWB in trauma-related massive transfusion protocols, particularly in prehospital and military scenarios. However, further in vivo validation and clinical testing are required before implementation.

Our study has certain limitations. Firstly, we limited the storage time of the samples to 7 days, as per the regulatory guidelines of the Russian Federation, which stipulate this as the maximum allowable period for the subsequent fractionation and cryopreservation of these blood units. This limitation was implemented to prevent the disposal of the studied units of preserved blood. Second, we utilized a small sample size of 12 individuals in each group; however, we believe this is sufficient to establish statistical differences between the groups on the designated control days. Only male donors were included in each group, which helps minimize risks that could affect the study results and data standardization [59]. The third limitation is that the results of in vitro studies cannot reliably predict the behavior of cells in vivo. Further research using labeled isotopes is necessary to address questions regarding the subsequent function and elimination of transfused cells from the body. At the moment, we cannot compare this with the Terumo Imuflex WB-SP platelet-sparing filter bag set because it is unavailable in Russia.

## 5. Conclusions

The obtained data provided a comprehensive assessment of the quality and safety of whole blood prepared using a modified technology combining partial leukoreduction and pathogen reduction. Our study demonstrates that leukoreduced, pathogen-reduced whole blood may serve as a potential alternative to conventional component therapy for patients with massive blood loss in prehospital and early hospital settings. While the infectious safety of pathogen-reduced blood is well established, its use in acute hemorrhagic settings remains underexplored. Our findings contribute to this field by showing preserved structural and functional properties of erythrocytes and coagulation profiles after the PR treatment, supporting the rationale for further in vivo validation.

While this study focused on morphological and mechanical properties, the findings provide a foundation for future investigation into the molecular pathways underlying membrane stability and cytoskeletal resilience during pathogen reduction and storage.

## Data Availability

The datasets used and analyzed during the current study are available from the corresponding authors upon request.

## Abbreviations

The following abbreviations are used in this manuscript:

AFM	Atomic force microscopy
E	Young’s modulus
PR	Pathogen reduction
PLTs	Platelets
RBCs	Red blood cells
WBCs	White blood cells
WB	Whole blood
